# Editorial: Cytokines and Pain

**DOI:** 10.3389/fimmu.2021.788578

**Published:** 2021-10-21

**Authors:** Larissa G. Pinto, Felipe A. Pinho-Ribeiro, Waldiceu A. Verri

**Affiliations:** ^1^ Wolfson Centre for Age-Related Diseases, King’s College London, London, United Kingdom; ^2^ Department of Immunology, Harvard Medical School, Boston, MA, United States; ^3^ Department of Pathology, Londrina State University, Londrina, Brazil

**Keywords:** pain, cytokine, neuro-immune communcation, microglia, astrocyte, interleukin, nociceptor, chemokine

Cytokines are released by immune and non-immune cells to orchestrate a great variety of physiological and disease processes. In special, cytokines can both directly and indirectly activate nociceptor sensory neurons modulating pain development. In turn, nociceptors also function as cellular regulators of the immune response in sterile and infectious diseases. All these aspects were within the aims of this Research Topic entitled “Cytokines and Pain”. There were two review articles and nine research articles published. Starting with the two reviews, they used different approaches. Vanderwall and Milligan discuss the contribution of cytokines to pain as molecules that link the immune and nervous system cells from peripheral tissues to the spinal cord (SC) triggering pain with the underlying contribution of neuroimmune interactions. Another focus of the review is the current gene therapies targeting cytokines to reduce pathological pain. Sexual dimorphism in pain and some additional inflammatory pathways are discussed. Santos et al. address the contribution of cytokines to nociceptor activation at four levels; in the peripheral tissues, at the dorsal root ganglia (DRG) in which the cellular bodies and nucleus of primary afferent neurons reside, and at spinal cord (SC) and supra-SC levels. The discussion approaches cellular interactions and role of cytokines to pain at each level. Their table one also brings a comprehensive view of cytokine families, cellular sources, and roles in pain and diseases.

We organized the nine research papers in this editorial from the periphery to the CNS. Napimoga et al. investigated the inflammatory and nociceptive effects of secreted osteoclastogenic factor of activated T cells (SOFAT), a novel cytokine, in collagen-induced arthritis (CIA). The intra-articular injection of 10 ng of SOFAT induced a four days-lasting mechanical hyperalgesia. SOFAT levels were higher in joint samples of CIA mouse than naïve mouse, and in synovial samples of rheumatoid arthritis patients than in osteoarthritis patients. Thus, bringing the first insight about the role of SOFAT in pain and inflammation in arthritis.

Guo et al. demonstrate that the injection of NGF in the trigeminal ganglia (TG) reduces the bite force upon tooth movement, a surrogate measure of pain. Inhibiting NGF enhanced the bite force, indicating reduced pain. NGF and tooth movement increased CGRP and CCL19 expression in the TG as well as CCL19 injection in the TG induced pain. Anti-CCL19 inhibited tooth movement pain and NGF enhanced tooth movement pain. Thus, demonstrating a novel role for CCL19 in tooth movement- and NGF-dependent pain.

Gonçalves et al. asked what was maintaining pain in a model of antigen-induced monoarthritis (AIA) after articular inflammation resolution. Mechanical hyperalgesia was accompanied by c-Fos activation in the DRG by the 8^th^ day when peripheral inflammation has already ended. At the DRG (L4 level) there was enhanced mRNA expression of TNF-α and TNFR2, and TNF-α levels. And etanercept (a soluble TNFR2) inhibited the persistent pain. Thus, DRG TNF-α and TNFR2 explain the persistent pain in AIA after peripheral inflammation resolution.

Patients with Chagas disease caused by *Trypanosoma cruzi* report pain in the acute phase of the disease. Borghi et al. demonstrate for the first time that pain during *T. cruzi* infection involves the activation of DRG neurons and SC microglia and astrocytes. There is interaction of SC glial cells and neurons that *via* NFkB control the expression of CX3CR1, TNF-α and IL-1β since the inhibition of glial cells reduces the expression of those chemokines and cytokines both at the DRG and SC. Thus, unveiling a previously unknown mechanism of pain in *T. cruzi* infection.

Fonseca et al. identified the cytokine IL-27 as an important endogenous mediator that counteracts the development of neuropathic pain following spared nerve injury (SNI). Peripheral nerve injury induces IL-27 expression in the SC and DRG that activates IL-27 receptor-expressing cells (macrophages, microglia, and astrocytes), thus, triggering the production of IL-10 and controlling the intensity of pain. Piotrowska et al. observed that CXCL3 and CXCR2 co-localize with neurons in the SC of naïve and chronic constriction injury (CCI) animals. And that microglia could also be a source of CXCL3 in CCI. NVP CXCR2 20 (a selective CXCR2 receptor antagonist) treatment reduced SC production of CXCL3 and satellite glial cells activation in the DRG. Primary microglial and astroglial cultures corroborated microglia would be the main source of CXCL3. Thus, CXCL3/CXCR2 signaling has a role in CCI neuropathic pain that can be targeted by NVP CXCR2 20.

Nerve damage is also a key feature of multiple sclerosis (MS), an extremely debilitating neurodegenerative disease that is often accompanied by the development of chronic neuropathic pain. By analyzing the electroencephalography (EEG) of patients with central neuropathic pain and comparing the results with those from patients without neuropathic pain, Krupina et al. identified changes in the power spectral density (PSD) that were specific signatures of patients with neuropathic pain. The authors observed alterations in spectral EEG patterns and peak frequencies of MS patients with neuropathic pain, including increased PSD for the theta, beta1, and beta2 frequencies in most regions of interest. This work demonstrates the existence of putative alterations in cortical communication that are specific for MS patients with neuropathic pain and that could be the basis of the sensory dysfunction observed in these patients. The mechanisms involved in MS were also evaluated in a model of experimental autoimmune encephalomyelitis (EAE). Xiao et al. observed that HMGB1 (high mobility group box 1 protein) induced shh (sonic hedgehog) release in astrocytes culture, through RAGE (receptor for advanced glycation end-products)-mediated JNK, p38, and STAT3 phosphorylation. Furthermore, HMGB1 also promotes shh in EAE, and shh treatment was able to alleviate the progress of EAE in mice. Thus, this study suggests HMGB1/shh has a protective role in EAE.

Neural pain is an important symptom in leprosy patients. In a retrospective study, Angst et al. assessed serum levels of cytokines in leprosy patients with or without pain as well as diabetic neuropathy pain. The authors observed an increased level of IL-1β, TNF, TGF-β and IL-17 in leprosy patients with neuropathic or nociceptive pain when compared with painless leprosy patients. Moreover, serum levels of IL-6 were increased in both leprosy and diabetic neuropathic pain patients, being an interesting target to control pain. IL-1β was identified as a key cytokine associated with neural leprosy pain when compared to diabetic neuropathic pain and can be used as a biomarker for patient follow-up.

Concluding, the two review papers, seven research papers using animal models and two clinical research papers addressed peripheral, DRG, SC and supra-SC mechanisms focusing on cytokines and neuro-immune interaction mechanisms in pain.

## Author Contributions

All authors listed have made a substantial, direct, and intellectual contribution to the work and approved it for publication.

## Funding

LP received funding from Versus Arthritis UK (grant 21522), Wellcome Trust UK (205006/Z/16/Z) and FAPESP/KCL Joint Grant (process number 2017/50419-9). WAVJ receives Brazilian grants from PPSUS funded by Decit/SCTIE/MS intermediated by CNPq with support of Fundação Araucária and SESA-PR (agreement 041/2017); Programa de Apoio a Grupos de Excelência (PRONEX) grant supported by SETI/Fundação Araucária and MCTI/CNPq, and Governo do Estado do Paraná (agreement 014/2017); and CNPq (#443180/2016-4; # 427946/2018-2; # 309633/2021-4; # 307186/2017-2).

## Conflict of Interest

The authors declare that the research was conducted in the absence of any commercial or financial relationships that could be construed as a potential conflict of interest.

## Publisher’s Note

All claims expressed in this article are solely those of the authors and do not necessarily represent those of their affiliated organizations, or those of the publisher, the editors and the reviewers. Any product that may be evaluated in this article, or claim that may be made by its manufacturer, is not guaranteed or endorsed by the publisher.

